# Early cessation of ceramic production for ancestral Polynesian society in Tonga

**DOI:** 10.1371/journal.pone.0193166

**Published:** 2018-02-23

**Authors:** David V. Burley, Sean P. Connaughton, Geoffrey Clark

**Affiliations:** 1 Department of Archaeology, Simon Fraser University, Burnaby, BC, Canada; 2 Inlailawatash Limited Partnership, North Vancouver, BC, Canada; 3 School of Culture, History & Language, Australian National University, Canberra, Australia; Institut Català de Paleoecologia Humana i Evolució Social (IPHES), SPAIN

## Abstract

Ancestral Polynesian society is the formative base for development of the Polynesian cultural template and proto-Polynesian linguistic stage. Emerging in western Polynesia ca 2700 cal BP, it is correlated in the archaeological record of Tonga with the Polynesian Plainware ceramic phase presently thought to be of approximately 800 years duration or longer. Here we re-establish the upper boundary for this phase to no more than 2350 cal BP employing a suite of 44 new and existing radiocarbon dates from 13 Polynesian Plainware site occupations across the extent of Tonga. The implications of this boundary, the abruptness of ceramic loss, and the shortening of duration to 350 years have substantive implications for archaeological interpretations in the ancestral Polynesian homeland.

## Introduction

The presence of ceramic vessels in the archaeological record typically is viewed as a technological development with high functionality, a technology concurrent with sedentism and agricultural production, and a marker for increasing cultural complexity. It seems aberrant, then, that a society could or would abandon a robust potting industry without apparent reason. Such was the case in the Polynesian homeland of Tonga where Lapita colonizer groups of 2850 cal BP produced a range of both decorated and plain ceramic vessel forms. Within a period of approximately 150 years, the decorated vessel forms disappeared completely with the potting industry then focused on a more limited range of undecorated types. Ultimately these ceased to be made, with ceramic manufacture absent in all Polynesian societies at the time of European contact. Kirch [1] rightfully notes that this type of ceramic sequence runs “… backwards from what we have become accustomed to seeing in most parts of the world”.

**Fig 1:**
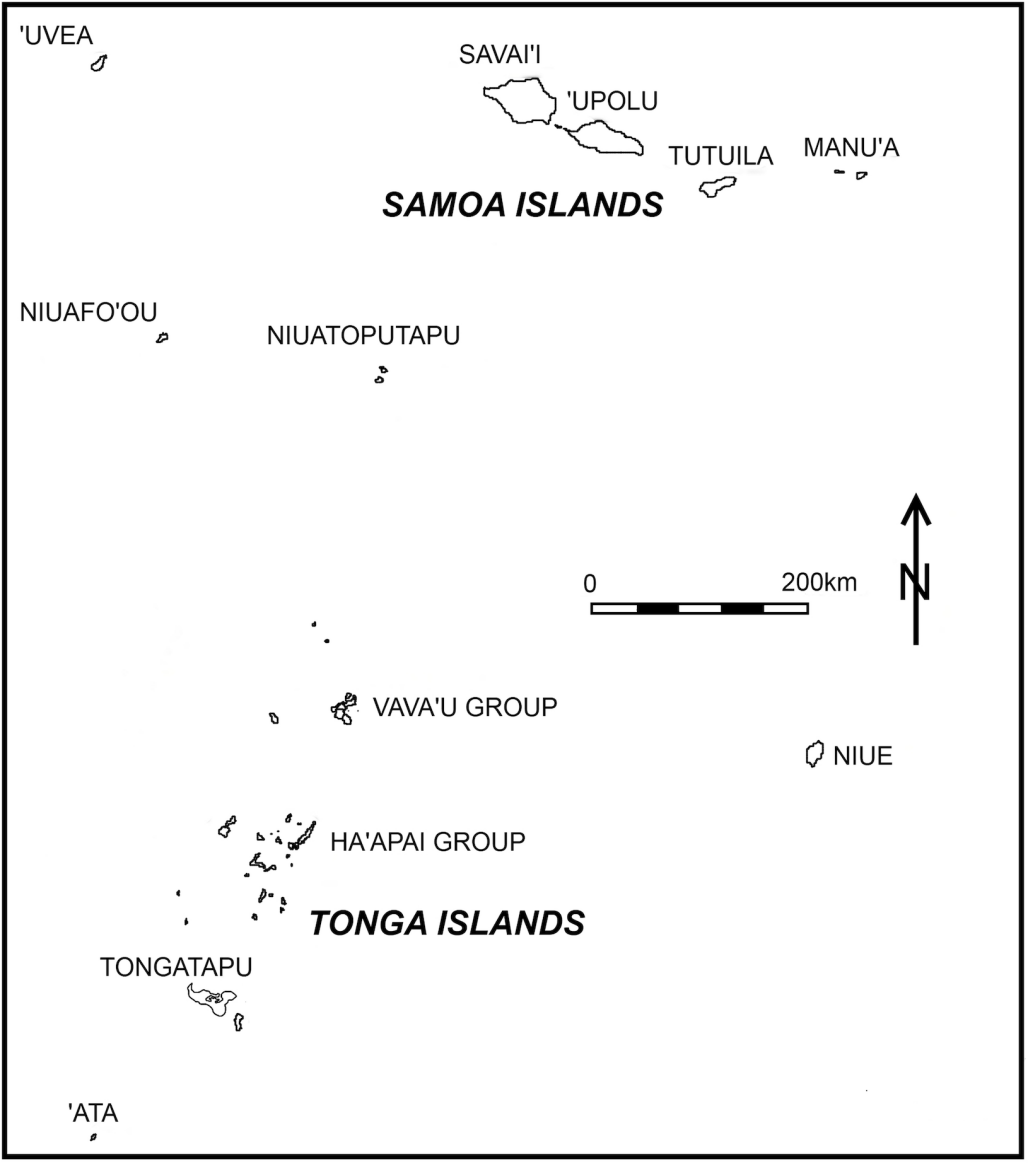
Map of islands within the ancestral, West Polynesian homeland. Two additional islands, Futuna and Alofi, are not included on the map being 300 km northwest of Niuafo’ou. The outliers of Niuafo’ou and Niuatoputapu are part of traditional and contemporary Tonga.

In this paper, we address the timing for ceramic production loss in the archaeological record of Tonga. The island of Tongatapu in southern Tonga was the first Polynesian island group to be colonized by Lapita peoples, and it is from here that initial settlement throughout most of the ancestral Polynesian homeland emanated (Fig 1). Our data incorporate a volume of 44 existing and new radiocarbon dates for the Polynesian Plainware phase in Tonga as recovered from 13 sites across the archipelago. The Polynesian Plainware phase is the post-Lapita temporal interval defined by plain ceramic wares but modeled as an instrumental period for the emergence of Polynesian society [2]. Rather than long term gradual loss of the industry where ceramic wares become increasingly simplified and degraded, or where the timing for ceramic loss across the Tongan group is varied, the data are uniform in illustrating this event to be early and contemporaneous throughout the archipelago. In contrast to most if not all current interpretations, the Polynesian Plainware phase is also short in duration. The implications of these results for our understanding of the conceptual framework for ancestral Polynesian society, the Tongan past, and Tonga’s position within western Polynesian antiquity are examined.

## Context

Tongan archaeology has a long history of archaeological practice with a critical emphasis placed on questions related to first settlement. Relative to this, Burley has been involved in a quarter century plus research program on Polynesian origins in Tonga where archaeological survey and excavations have been carried out within each of the three principal island groups–Tongatapu, Ha’apai and Vava’u. Initial landfall by people with distinctive Lapita ceramics took place ~ 2850 cal BP at the entrance to the lagoon system off the northeast coast of Tongatapu [3], [4]. High precision dates from U/Th measurement of *acropora* coral tools, AMS radiocarbon dates on short-lived wood charcoals and Bayesian modeling with other radiocarbon dates provide clear insight into the timing of subsequent population movements [5]. After a lag interval on Tongatapu of 70–90 years, occupation expansion into the central Ha’apai islands occurred between 2772 and 2759 cal BP with almost simultaneous movement northward into the Vava’u group between 2805 and 2760 cal BP. We presume Lapita occupation of the far northern outlier of Niuatoputapu and Samoa were extensions of this movement. Importantly for this paper, Bayesian modeling of dated samples provide an estimated duration for the Lapita ceramic phase in each group. This includes end dates on Tongatapu of 2703–2683 cal BP (duration 129–158 years), on Ha’apai of 2728–2716 cal BP (duration 32–49 years) and on Vava’u of 2709–2680 cal BP (duration 51–82 years)[5]. The end dates simultaneously define the beginning of the sequent Polynesian Plainware phase for each group, hereafter generalized as 2700 cal BP.

The addition of Polynesian in reference to a post-Lapita phase characterized by undecorated ceramics in Tonga and Samoa is attributable to Green [6], [7]. In keeping with arguments by Groube [8] that Polynesians did not come from anywhere but “*becam*e” Polynesian, Green equated the onset of Polynesian ethnogenesis with the loss of decorated Lapita wares. Polynesian was added to plain wares to denote this association. Kirch and Green [2] provide a substantive analysis of ancestral Polynesian culture, integrating archaeology, linguistics, ethnographic comparison and biological anthropology. Polynesian Plainware ceramics continue to be identified as important correlates within this process, particularly as regional variations in ceramic assemblages might reflect upon linguistic divergences between island groups throughout West Polynesia. Kirch and Green [2] further interpret the end of pottery manufacture as concomitant with “the break-up of Ancestral Polynesian culture” and the movement of groups into the Polynesian outliers and central East Polynesia. In the Kirch and Green [2] scenario, the Polynesian Plainware phase would have substantial time depth extending from terminal Lapita into at least the initial centuries of the Christian era. The duration of the Polynesian Plainware phase in different areas across West Polynesia is expected to be varied; in some areas pottery production is argued to have continued well into later prehistory [9], [10]. Connaughton [11] acknowledges this in his regional review of Polynesian Plainware phase dates for western Polynesia but goes on to project a general duration lasting 1200–1300 years after the loss of decorated vessels.

**Fig 2:**
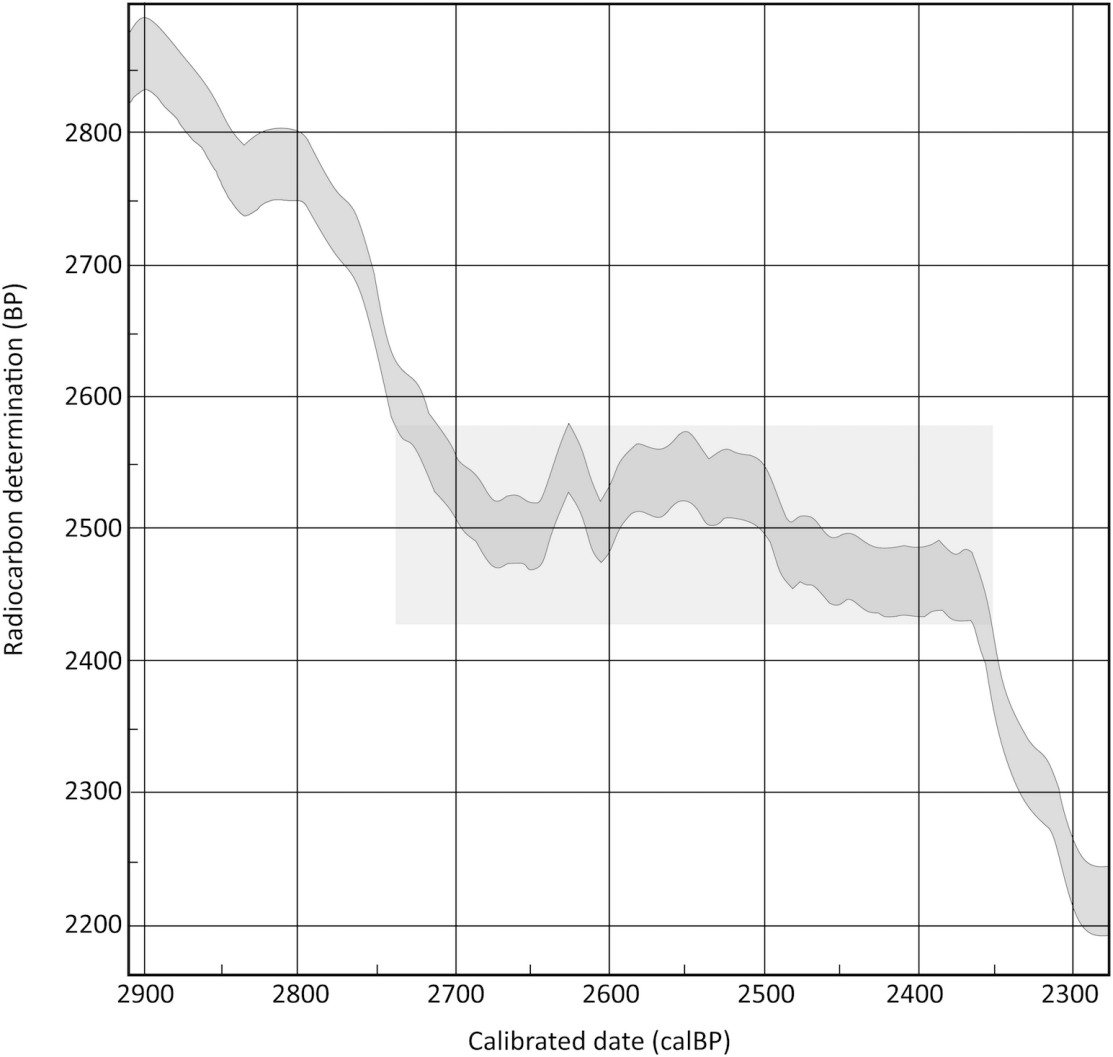
The Hallstatt Plateau (shaded) as constructed on the OxCal radiocarbon calibration program for the southern hemisphere 2013 (SHcal13) calibration curve [13].

The Bayesian analysis of Tongan ^14^C and U/Th dates described above was successful in production of a high precision chronology for first Lapita settlement and expansion. A similar effort to apply Bayesian modeling to then existing Polynesian Plainware dates was less rewarding. Very few acceptable Plainware dates existed for Tongatapu (n = 3) or Vava’u (n = 2) and all dates, including those for Ha’apai (n = 14), fell within a segment of the radiocarbon calibration curve referred to as the Hallstatt Plateau [12]. The latter is a flattening in the curve that homogenizes calibration outputs to a roughly 300-year interval (Fig 2). The Ha’apai dates did imply a short duration for the Polynesian Plainware phase. Skepticism over their accuracy nevertheless left “open the question of a boundary end” [5]. This skepticism was in part a concern that the Ha’apai dates were skewed in sample selection for documentation of the Lapita to Polynesian Plainware transition. Skepticism also was rooted by existing thought that the Polynesian Plainware phase was of longer duration and potentially varied in its chronology among island groups across Tonga and elsewhere in western Polynesia.

## Methods, radiocarbon samples and dates for the Polynesian Plainware phase

All necessary permits were obtained for the described study, which complied with all relevant regulations. Permits were approved by Cabinet Decision (CD) of the Government of Tonga including CD 996 July 23, 1990; CD 868 June 20,1996; CD 395 March 26, 1997; CD 660 May 5, 1999; CD 1259 August 3 2001 and CD 527, April 2, 2003. Archaeological collections recovered as part of fieldwork are reposited and accessible within the Department of Archaeology at Simon Fraser University (Burley), Burnaby, BC, Canada, www.sfu.ca/archaeology or in the School of Culture, History & Language, Australian National University (Clark), Canberra, Australia, http://chl.anu.edu.au/.

To address the extent of Polynesian Plainware chronology for Tonga more thoroughly, we add 25 additional radiocarbon measurements to the sample of 19 dates previously analyzed. Detailed information for the 44 samples is provided in S1 File. Sixteen of these samples heretofore have yet to be reported upon. All dates are taken from wood charcoal with 17 from short-lived materials, predominantly coconut endocarp. Intentionally we exclude dates on marine shell and bone for reasons provided in Burley et al. [5]. Sample distribution now includes 14 dates from five sites on Tongatapu, 24 dates from six sites in the Ha’apai group and six dates from two sites in Vava’u. The skew in spatial distribution relates to number of investigated sites and availability of charcoal samples for dating. In the Ha’apai and Tongatapu sites charcoal samples are abundant; in Vava’u they are more limited in their occurrence. Notably all but two of the samples were recovered and submitted by Burley or Clark for direct measurement of ^14^C with accelerator mass spectrometry.

The 13 archaeological sites from which the samples derive are consistent in being coastal settlements first occupied during or near the end of the Lapita phase but with continuity through Polynesian Plainware into later aceramic periods; several have continuity into contemporary villages. Exceptions are Holopeka on Lifuka Island in Ha’apai and Fakala’a and Moisa on the Fangakakau Lagoon shore of Tongatapu. In these sites occupation did not begin until the Polynesian Plainware phase, but with settlement continuing up to the present. All sites have defined stratigraphic contexts within which attempts are made to infer later, middle or earlier Polynesian Plainware contexts for many of the samples. In most cases the Polynesian Plainware stratum is homogenous in its composition having a matrix of organically rich sediment, abundant ceramics and other cultural materials, faunal remains including shell fish and fire broken rock from hearth or processing features. To differentiate earlier from later Polynesian Plainware samples, therefore, relies on vertical provenience either maintained by depth of sample or the spit the sample was recovered from. This is not an ideal context for relative chronology as occupation deposits for the Polynesian Plainware stratum may vary in depth and/or thickness across a site while midden accumulation is irregular in its deposition. In three cases, we are able to date multiple samples at varying depths/spits from within single 1 x 1 m excavation units. These units provide a tentative measure of vertical control against which dates can be assessed. The nature and abundance of the undecorated ceramic assemblage associated with radiocarbon samples also has potential for information on relative age [14].

**Fig 3:**
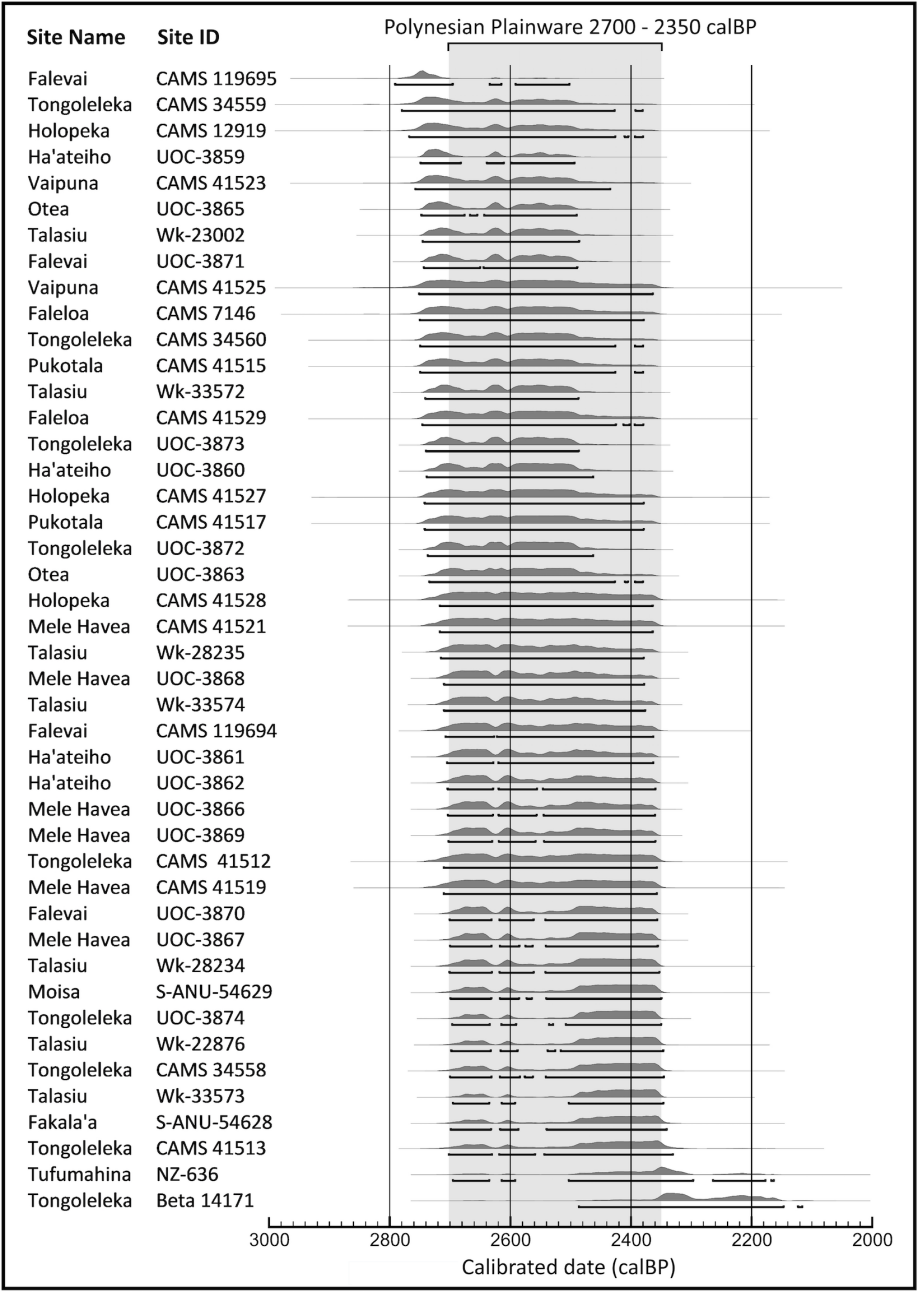
Plot of calibrated radiocarbon ages (95.4%) for Polynesian Plainware sites in Tonga. Plot was done on Oxcal with calibrations done using the southern hemisphere 2013 (SHcal13) calibration curve [14].

Table 1 provides measurements for the 44 samples with calibrated ranges plotted on Fig 3. Expanded detail for individual dates is incorporated as S1 File. All samples, including those previously reported, have been calibrated or recalibrated employing the SH13 southern hemispheric calibration curve with 68.2% and 95.4% probability ranges [13]. The cumulative result can only be described as consistent, homogeneous, informative and convincing. The earliest uncalibrated radiocarbon date, 2645±35 BP (CAMS 119695), is from the site of Falevai on Kapa Island in the Vava’u island group. The most recent are 2330±60 BP (Beta 14171) from Tongoleleka on the island of Lifuka in the Ha’apai group and 2380 ± 51 BP (NZ 636) from Tufumahina on the island of Tongatapu. The stratigraphic context for the Falevai sample marks the Lapita to Polynesian Plainware transition and the calibrated age (2791–2502 cal BP, 95.4%) is appropriate to the event being dated [5]. The Tongoleleka and Tufumahina dates are the only two samples within the data set without AMS measurement, and they are the only two samples not recovered and selected for dating by either Burley or Clark. That they are the most recent ^14^C measurements may be coincidence, but a circumstance potentially identifying them as outliers. The calibrated ranges for these dates at 95.4%, however, overlap with other samples in the data set leaving us to include them here.

**Table 1:** Radiocarbon dates^a^ for Polynesian Plainware occupation strata, Kingdom of Tonga ordered by oldest to youngest.

Group/Island^b^	Site	Sample ID	14C date	Cal BP 95.4%^c^	Material	Reference
Vava'u/Kapa	Falevai	CAMS 119695	2645 ± 35	2791–2502	charcoal	[14]
Ha'apai/Lifuka	Tongoleleka	CAMS 34559	2600 ± 60	2780–2380	charcoal	[5]
Ha'apai/Lifuka	Holopeka	CAMS 12919	2590 ± 60	2768–2380	charcoal	[5]
Tongatapu	Ha'ateiho	UOC-3859	2583 ± 22	2749–2494	endocarp	new
Ha'apai/'Uiha	Vaipuna	CAMS 41523	2580 ± 50	2758–2434	charcoal	[5]
Vava'u/Kapa	Otea	UOC-3865	2572 ± 26	2748–2490	charcoal	new
Tongatapu	Talasiu	Wk-23002	2562 ± 30	2746–2486	charcoal	[15]
Vava'u/Kapa	Falevai	UOC-3871	2561 ± 25	2744–2489	charcoal	new
Ha'apai/'Uiha	Vaipuna	CAMS 41525	2560 ± 80	2752–2364	charcoal	[5]
Ha'apai/Foa	Faleloa	CAMS 7146	2560 ± 60	2750–2379	charcoal	[5]
Ha'apai/Lifuka	Tongoleleka	CAMS 34560	2560 ± 50	2750–2380	charcoal	[5]
Ha'apai/Ha'ano	Pukotala	CAMS 41515	2560 ± 50	2750–2380	charcoal	[5]
Tongatapu	Talasiu	Wk-33572	2553 ± 25	2741–2487	endocarp	[15]
Ha'apai/Foa	Faleloa	CAMS 41529	2550 ± 50	2746–2380	endocarp	[5], [16]
Ha'apai/Lifuka	Tongoleleka	UOC-3873	2550 ± 23	2740–2486	charcoal	new
Tongatapu	Ha'ateiho	UOC-3860	2542 ± 25	2738–2462	charcoal	new
Ha'apai/Lifuka	Holopeka	CAMS 41527	2540 ± 50	2742–2379	endocarp	[5], [16]
Ha'apai/Ha'ano	Pukotala	CAMS 41517	2540 ± 50	2742–2379	charcoal	[5]
Ha'apai/Lifuka	Tongoleleka	UOC-3872	2540 ± 24	2737–2462	charcoal	new
Vava'u/Kapa	Otea	UOC-3863	2529 ± 29	2734–2380	endocarp	new
Ha'apai/Lifuka	Holopeka	CAMS 41528	2510 ± 50	2717–2364	charcoal	[5]
Ha'apai/Ha'afeva	Mele Havea	CAMS 41521	2510 ± 50	2717–2364	charcoal	[5]
Tongatapu	Talasiu	Wk-28235	2510 ± 30	2715–2379	endocarp	[15]
Ha'apai/Ha'afeva	Mele Havea	UOC-3868	2505 ± 22	2710–2378	endocarp	new
Tongatapu	Talasiu	Wk-33574	2504 ± 25	2710–2376	endocarp	[15]
Vava'u/Kapa	Falevai	CAMS 119694	2500 ± 35	2708–2363	charcoal	[16]
Tongatapu	Ha'ateiho	UOC-3861	2499 ± 22	2705–2363	charcoal	new
Tongatapu	Ha'ateiho	UOC-3862	2493 ±25	2704–2360	charcoal	new
Ha'apai/Ha'afeva	Mele Havea	UOC-3866	2493 ± 22	2704–2360	charcoal	new
Ha'apai/Ha'afeva	Mele Havea	UOC-3869	2491 ± 22	2702–2360	endocarp	new
Ha'apai/Lifuka	Tongoleleka	CAMS 41512	2490 ± 51	2710–2357	endocarp	[5], [16]
Ha'apai/Ha'afeva	Mele Havea	CAMS 41519	2490 ± 50	2710–2357	endocarp	[5], [16]
Vava'u/Kapa	Falevai	UOC-3870	2483 ± 22	2700–2356	charcoal	new
Ha'apai/Ha'afeva	Mele Havea	UOC-3867	2478 ± 22	2700–2356	unid nut	new
Tongatapu	Talasiu	Wk-28234	2473 ± 31	2701–2353	endocarp	[15]
Tongatapu	Moisa	S-ANU-54629	2461 ± 32	2700–2350	endocarp	new
Ha'apai/Lifuka	Tongoleleka	UOC-3874	2460 ± 22	2696–2350	charcoal	new
Tongatapu	Talasiu	Wk-22876	2452 ± 30	2698–2346	charcoal	[15]
Ha'apai/Lifuka	Tongoleleka	CAMS 34558	2450 ± 40	2700–2346	charcoal	[5]
Tongatapu	Talasiu	Wk-33573	2448 ± 25	2695–2346	endocarp	[15]
Tongatapu	Fakala'a	S-ANU-54628	2439 ± 38	2700–2431	endocarp	new
Ha'apai/Lifuka	Tongoleleka	CAMS 41513	2430 ± 50	2702–2330	endocarp	[5], [16]
Tongatapu	Tufumahina	NZ-636	2380 ± 51	2695–2163	charcoal	[17]
Ha'apai/Lifuka	Tongoleleka	Beta 14171	2330 ± 60	2487–2116	charcoal	[11]

^a^Detailed data for dates are given in S1 File.

^b^ Island groups on which sites are located are identified in Fig 1.

^c^Dates are calibrated with Oxcal using the southern hemisphere 2013 (SHcal13) calibration curve [14].

In the southern hemisphere calibration curve, the Hallstatt Plateau can be plotted between 2430 and 2585 BP (Fig 2) but with variable impacts on earlier and later dates based on standard error and Gaussian distribution (Fig 3). Significantly, all but five dates within the sample fall directly within the 2430–2585 BP interval creating substantive problems for precision in calibration or interpretation of regional variation in chronology. That being said, we are able to constrain the earliest boundary for the Polynesian Plainware phase to ca 2700 cal BP based on end dates for the Lapita phase in previous Bayesian analysis [5]. We also feel secure in identifying its most recent boundary as no more than 2350 cal BP, the approximate calibrated end date for the Hallstatt Plateau [12]. The duration of ceramic production after the disappearance of Lapita pottery across all of the island groups in Tonga, thus, has an estimated chronological extent of 350 years or potentially less.

Trepidation related to age skewing in sample selection for Ha’apai dates and possible problems related to chronological associations for stratigraphic context are noted earlier. The three excavated 1 x 1 m units with multiple radiocarbon dates at increasing depths across the Polynesian Plainware stratum were intended to provide perspective in this regard (Table 2). The samples derive from a single unit at Talasiu excavated by Clark [15] and two units from Tongoleleka on Lifuka Island, Ha’apai excavated by Burley [16]. In each case, the radiocarbon samples were selected for their varied depths within the Polynesian Plainware phase occupation zone and, based on depth, are given contextual associations of lower to upper at Talasiu or Lapita/Plainware transition to Late Plainware at Tongoleleka. As illustrated in Table 2, in no case is there a progressive sequence of dates. This is not surprising nor contradictory. All dates are influenced by the Hallstatt Plateau which is caused by atmospheric variations in ^14^C content and changes in the carbon cycle [18]. The consequence is a flattening in the curve but with undulating wiggles of peaks and troughs (Fig 2). A date could fall in the lower trough of a wiggle where there is a decrease in atmospheric ^14^C while another may fall on a peak where it is increased. The consequence, then, can be the appearance of a reversed ^14^C clock as is the case at Talasiu and Tongoleleka Unit 11.

**Table 2:** Sequential radiocarbon dates for Polynesian Plainware occupations from three excavation units in the Kingdom of Tonga.

Site/Unit	Spit/Depth	Context	^14^C Date	Cal 95.4%	Lab Number
Talasiu Pit 2	Spit 4	upper	2553 ± 25 BP	2741–2487 BP	Wk-33572
Talasiu Pit 2	Spit 11	middle	2448 ± 25 BP	2695–2346 BP	Wk-33573
Talasiu Pit 2	Spit 18	lower	2504 ± 25 BP	2710–2376 BP	Wk-33574
Tongoleleka Unit 11	Level 4 (36 dbs)^a^	mid to late PPW^b^	2540 ± 24 BP	2737–2462 BP	UOC-3872
Tongoleleka Unit 11	Level 4 (38 dbs)	mid to late PPW	2490 ± 51 BP	2710–2357 BP	CAMS 41512
Tongoleleka Unit 11	Level 7 (66 dbs)	early to mid PPW	2430 ± 50 BP	2702–2330 BP	CAMS 41513
Tongoleleka Unit 11	Level 9 (89 dbs)	LA^c^/PW Trans	2460 ± 22 BP	2696–2350 BP	UOC-3874
Tongoleleka Unit 4	Level 5 (50 dbs)	mid PPW	2450 ± 40 BP	2700–2346 BP	CAMS 34558
Tongoleleka Unit 4	Level 8 (75 dbs)	early PPW	2600 ± 60 BP	2780–2380 BP	CAMS 34559
Tongoleleka Unit 4	Level 10 (103 dbs)	LA/PPW Trans	2560 ±50 BP	2750–2380 BP	CAMS 34560

^a^dbs = depth below surface

^b^PPW = Polynesian Plainware

^c^LA = Lapita

The implication of the Hallstatt plateau for radiocarbon calibration is significant in that chronological precision becomes all but impossible. This is particularly true in Europe where almost the entirety of Iron Age settlement falls within its temporal span [19]. A partial solution has been wiggle-matching, a technique that matches the shape of a series of sequentially calibrated radiocarbon ages to the shape of the radiocarbon calibration curve [20]. This requires known age separations between dates to accurately fit the curvSe, limiting most applications to wood samples where a tree ring sequence is present. Alternatively, where depth measurements for samples provide chronological order, matrix accumulation rates must be constant and known [21]. Neither of these applies to current considerations of the Polynesian Plainware phase dates where virtually all samples are small flecks of charcoal or where accumulation rates in midden contexts are assumed erratic. The potential for refinement of a Polynesian Plainware chronology exists nevertheless. This will require future acquisition and U/Th dating of coral artifacts from appropriate contexts, as has been employed in earlier Bayesian analysis of Lapita phase settlement expansion across Tonga [5]. U/Th measurement on coral is not affected by fluctuation in atmospheric carbon while calibration is independent of radiocarbon calibration curves. U/Th measurement also provides 2 σ calibrated dates with exceptional precision in the range of ± 6 to 10 years for the temporal period under consideration.

## Discussion

The Polynesian Plainware phase across western Polynesia is defined as a pivotal transition marking the onset of ancestral Polynesia. The 44 radiocarbon dates for this phase in Tonga as presented here are categorical and informative. Coming from 13 sites across multiple islands from south to north, these delineate a maximum temporal interval of 2700–2350 cal BP for its duration. The most recent boundary for this interval is substantially earlier than previously inferred for cessation of ceramic production in Tonga; indeed, it exceeds our prior estimates by at least 800 years [11], [22]. Compressing existing interpretations of development and change during the Polynesian Plainware phase into a 350-year temporal duration has several implications for an understanding of ancestral Polynesian society as it has been previously defined, the Tongan past, as well as regional relationships in western Polynesia. Each of these issues is taken up as final discussion.

### Ancestral Polynesian society

The settlement of Polynesia represents a final phase in a rapid dispersal of peoples across Remote Oceania defined by Lapita ceramics as has been noted. Once western Polynesia was colonized, further movement eastward into the remainder of Polynesia did not occur for as much as 1800 years [23]. Referred to as the “long pause”, this was a period of time in which Polynesians literally became Polynesian, developing a Polynesian cultural template as well as a discrete linguistic sub-stage, proto-Polynesian. It is a developmental phase typically referred to as ancestral Polynesia with western Polynesia being the ancestral Polynesian homeland [2]. The appearance of Polynesian Plainware ceramics as a distinct and integrated assemblage is taken as a marker for its earliest beginnings, one distinguishing it from Lapita in western Polynesia and elsewhere in Oceania.

Kirch and Green [2] argue that Polynesian cultures form a phyletic unit to which a phylogenetic approach may be applied. All Polynesians, in their view, share a common history and a common ancestor. Through detailed analyses of the data, not only can that history be mapped as a series of diverging relationships, but the ancestral cultural template can be appropriately defined through integration of comparative historical linguistics, comparative ethnography, archaeological data, and to a lesser extent biological anthropology. Grounded in proto-Polynesian lexical reconstruction, this approach has provided a powerful tool for archaeological inference of the Polynesian Plainware phase. Numerous aspects of material culture, social organization, ritual activities and the like are poorly if ever preserved in archaeological context but quite accessible through a proto-Polynesian lexicon or cross-referenced in comparative ethnography through homologous relationship. In this Kirch and Green [2] go so far as to propose proto-Polynesian terms potentially applicable to Polynesian Plainware ceramic types.

Proto-Polynesian is defined as an innovation-rich language stage requiring a considerable period of time in its elaboration [2]. Pawley [24], for example, estimates a developmental period of 1000 years during which western Polynesian peoples spoke the same language and where a shared collective of linguistic innovations structured and came to define proto-Polynesian. To equate it with the Polynesian Plainware phase similarly implies a lengthy period of common development across western Polynesia as a whole. That we define the end of ceramic production in Tonga at 2350 cal BP complicates this matter. As a homogenous entity for ancestral Polynesia, this phase now has a maximum extent of no more than 350 years and quite possibly less. The development of regional variations in Polynesian Plainware ceramic forms similarly has been correlated with emergent linguistic and societal diversity in the different island groups of western Polynesia [2]. A glotto-chronological estimate for the breakup of proto-Polynesian [24], an event presumably tied to typological divergence in the archaeological record, also is discordant in that it now post-dates Polynesian Plainware in Tonga by 300 years.

The correlation of linguistic and archaeological evidence in Polynesia is challenging at best since each form an independent data set. Material culture and language can be subject to change for different reasons, and change in each can occur at different rates. A direct correlation between the Polynesian Plainware phase as defined by ceramics and a developmental sequence for proto-Polynesian language justifiably can be queried. This circumstance does not contest the validity of comparative historical linguistics. For the linguist, the absence of archaeological correlation makes it more difficult to position linguistic events within real time chronology that archaeologists are able to generate. For the archaeologist, it dampens our abilities to fill in details of the past that are not well preserved in the archaeological record. It does not, however, obviate insights gained from lexical reconstruction where archaeological data may be able to test those inferences.

### Implications for Tongan demography

Beginning with first land fall on Tongatapu, the Lapita settlement of Tonga was expansive throughout the archipelago yet ephemeral in population size and the number of settlements involved in this undertaking [25]. All Lapita sites were positioned in coastal settings for the exploitation of foreshore and marine resources but with subsistence economy additionally incorporating low level horticultural production [4], [11]. The full duration of this phase on Tongatapu is only 150 or so years and considerably less elsewhere [5]. At best, Lapita settlement provided a nascent foothold for subsequent events in the Polynesian Plainware phase.

Polynesian Plainware phase sites in Tonga are ubiquitous, sometimes substantial, and they occur on virtually every inhabitable island across the archipelago, including both coral limestone and volcanic formations [26]. The abundance and distribution of these sites suggest a period with major demographic growth and, by the end of the phase, an expansive if not full use of the Tongan landscape [11]. On Tongatapu, Plainware settlement was not restricted to the leeward lagoon system as was the case with most sites of the Lapita phase. Rather sites are found in all coastal areas, on small offshore islands and they are dispersed across the island’s interior [25]. Groube [8], in fact, comments that plainware pottery concentrations are so dense in places, that Tongans considered ceramic sherds to be “part of the soil itself”. On the island of Lifuka, in the central Ha’apai group, a ridge of occupation with Plainware ceramics is present on the leeward coast stretching over a continuous distance of 5 km, but with several intensive occupation nodes [25]. And even on the far northern outlier of Niuatoputapu, Kirch reports a 50m wide zone of largely Plainware archaeological deposits that encircle the island’s volcanic core [27].

This distribution and density of Plainware sites suggests a period of population growth far beyond what might be expected for the 350 years of the Polynesian Plainware phase. If we employ Hassan’s [28] maximum growth rate of 0.0052 for prehistoric populations, assume a Lapita founder colony of 100 individuals with exponential growth, and calculate a population at 2350 cal BP for the Polynesian Plainware phase, the projected number of individuals is no more than 1337 (S2 File). This number appears incongruous to the archaeological record of Tongatapu alone; with other islands throughout the archipelago included, it is completely unfeasible. Since there is no archaeological indication of largescale immigration into Tonga during the Polynesian Plainware phase, a far more robust growth rate must have been at play. This situation has been predicted for the early settlement of Remote Oceania, where founder colonies are not subject to density dependent controls [1]. In the case of pre-European Maori peoples in New Zealand, for example, the population growth rate is calculated as 0.00875 based on studies of skeletal remains [29]. If this rate is applied to early Tonga as above, the population size at the end of the Polynesian Plainware phase is projected to be 7794 individuals, a number more consistent with the archaeological record as described.

Exponential population growth ultimately is constrained by density dependent controls unless an upward shift in carrying capacity occurs through environmental change or technological innovation [30]. In Tonga, species extinctions, extirpations and resource compression occurred in the Lapita phase [31], serving to reduce carrying capacity limits. To accommodate and support further population growth, we believe a fundamental shift took place in subsistence economy at the end of the Lapita period. Polynesian Plainware phase site distributions across the Tongan landscape, including island interiors, not only anticipate greater reliance on agricultural production but represent a critical transformation to dry land agricultural practices if not field systems as is the case today [11]. This shift is far earlier than previously considered.

### Western Polynesian relationships or lack thereof

We have addressed the theoretical issues of ancestral Polynesian society in western Polynesia to the degree that it can be equated with the Polynesian Plainware phase. Assessing the implications of ceramic loss in Tonga at 2350 cal BP as it may relate to specific events or interpretations of the archaeological record in other areas of the Polynesian homeland is more difficult. This difficulty in part is due to limited data and/or interpretive frameworks for the Polynesian Plainware phase on other western Polynesian islands [11]. In the case of Samoa there also is ongoing debate on the extent, nature and timing for sustained settlement within the archipelago as a whole.

In the very broadest sense of comparison, ceramic sequences on other western Polynesian islands appear in tandem with that in Tonga. Limited assemblages of eastern Lapita ceramics characterize the earliest settlement episodes albeit secure radiocarbon dates to position these in time are few [11]. The suite of highly simplified decorative motifs on Lapita ceramics, and other decorative applications, suggest a later colonization event in a relative sense to Tonga. Accordingly, the exploration and expansion of Lapita peoples from Tongatapu northward through the islands of Ha’apai and Vava’u [5] potentially, if not probably, extended into these areas. As in Tonga, the Lapita period elsewhere in western Polynesia is brief with its end marked by loss of ceramic decoration.

The Polynesian Plainware ceramic assemblage in Tonga is well defined by Connaughton [11]. In comparison to Lapita ceramics, decoration is lost, as are some of the complex vessel forms and elaborate features (carination, sharply everted rims) to which it was applied. There is, nevertheless, direct continuity between undecorated ceramics in the Lapita phase and those continuing to be manufactured during the Polynesian Plainware phase. There also is diversity in vessel forms and sizes including jars, bowls and cups. This pattern of ceramic transition occurs on ‘Uvea [32], but it is not the case for either Futuna or on the various islands of Samoa. Rather, a limited range of vessels are present with either jars or bowls respectively dominant [11]. Regional variation in Polynesian Plainware ceramics across western Polynesia occurs at the beginning of this phase, not later as was implied in discussions of ancestral Polynesia. Equally notable, ceramic production on these islands is argued to continue long after 2350 cal BP; in the Samoa situation, substantially longer [9].

The case of Samoa is of particular note relative to both its settlement history and ceramic variability in comparison to the Polynesian Plainware phase in Tonga. Samoa in some respects is a geographic extension of Tonga positioned at the northern end of a natural sailing corridor along a 1000 km southwest to northeast axis (Fig 1). One might expect Samoa’s early culture history to be interwoven with Tonga, especially given Samoa’s less than 400 km distance from Niuatoputapu, a northern Tongan outlier with a settlement and ceramic sequence typical of Tonga [27]. Sites relating to first settlement of Samoa, however, are strikingly sparse and, for the Polynesian Plainware phase, strikingly different. The Lapita phase in Samoa is represented only by a single site on the island of ‘Upolu, Mulifanua. Mulifanua is submerged resulting from coastal subsidence, leading some archaeologists to conclude a similar fate for other Lapita settlements [33]. This interpretation more recently has been challenged [34]. Substantive research into shoreline geomorphology [35] suggest there to be highly restricted coastal plains on most islands while modern shoreline features did not occur until after 2000 BP. The Samoan landscape seems ill-suited to settlement expansion, with colonization limited to but a few isolated groups [36].

Polynesian Plainware settlement predating 2500 BP in Samoa is also sparse, suggesting to some [37] a partial abandonment of these islands following the Lapita period at Mulifanua. Bayesian analysis of radiocarbon and U/Th dates from recently excavated sites on Ofu island of the Manua group, nevertheless, convincingly document a Plainware settlement there occurring between 2717–2663 cal BP (68.2%)[38]. The Ofu ceramic assemblage has yet to be described in detail. Until that occurs, direct comparisons with Tonga and other sites in Samoa cannot be made. Questions of settlement continuity from Mulifanua, the speed and nature of ceramic change, or whether the Samoan archipelago was abandoned and resettled by separate migration events in the Plainware phase will remain unaddressed.

### Concluding notes on the question of ceramic loss

Our introductory remarks make note of the unusual circumstance for ancestral Polynesia where a rich ceramic tradition brought by initial Lapita colonists is completely lost by the end of the Polynesian Plainware phase. Ceramic vessels are not only highly functional in their use for cooking, storage and serving, but oftentimes serve as trade commodities and/or become integrated into the social or ritual fabrics of past societies [39]. Where agricultural production is central to subsistence economy, and where settlements are sedentary in nature, ceramics typically constitute a core component of material culture. Polynesian societies are a principal exception. As Davidson [40] comments some four decades ago, the loss of ceramic manufacture in western Polynesia and the failure of later Polynesians “to relearn the art” is an intriguing reflection of cultural processes requiring further study.

One of the earlier explanations for an absence of ceramics in Polynesian societies is that suitable clays or tempers required for production are difficult to acquire [41]. The robust nature of the ceramic industry in both Lapita and Polynesian Plainware periods in Tonga, if not elsewhere in western Polynesia, rules this out. More recent hypotheses center on functional questions of ceramic use, contesting the need for pottery production in light of alternative possibilities. Historic and ethnographic accounts of traditional Polynesia, it is emphasized, illustrate culinary practices, food service and storage without the requisite presence of ceramics [23], [42]. An absence of necessity, thus, provides the causal explanation for ceramic loss, the circular reasoning of the argument notwithstanding. And in combination, it has been claimed, the value of the ceramic industry had substantially diminished in the Polynesian Plainware phase, as decorated Lapita vessels were no longer produced as prestige goods or tradewares [23], [43]. That Lapita ceramics in Tonga were ever trade-wares, however, is questionable [44].

Green [7] highlights the social aspect of ceramic use in discussion of pottery loss in Samoa. He suggests changes in the social role of pottery, combined with functional considerations, hold most promise for understanding ceramic disappearance. The social context of ceramics has been raised by other authors, with implications for gender relations [43] or changes in the social prominence of potters themselves [45]. None of the explanations are adequately developed, nor supported by data, but they hint at processes difficult to identify through archaeology alone. The importance of a social role for pottery and its manufacture is reinforced and expanded upon in recent ethno-archaeological study of a potting community on Kadavu Island in Fiji [46]. The manufacture of ceramics within this community is not undertaken by a series of independent potters. Rather it is done as a collective, adhering to a set of ritualized and rigid protocols for individual manufacturing stages, from acquisition of clays and tempers to the final firing process. The protocols integrate the community, requiring co-operative relations between competing land holding groups while rationalizing manufacturing processes and relationships in mythologized/traditional narrative. It is hard to justify the application of a Kadavu analogue to an almost three millennium earlier potting community in western Polynesia. Yet the analogue is clearly insightful in its illustration of complex and impossible to recognize cultural behaviors that could well be embedded within ancestral Polynesian pottery production. Combined with a changing landscape of social relations and competitive interests during a period of rapid population growth and transformation within subsistence economy provides additional possibilities for abandonment of the ceramic industry.

Existing explanations for the early cessation of ceramic manufacture in Tonga, as described, are at best inferential hypotheses based for the most part in speculation. Whether archaeologists will ever be able to definitively unravel the how and why of ceramic loss in ancestral Polynesia seems unlikely. What we can say with certainty is that pottery loss was a simultaneous event across Tonga without evidence for a progressive degradation within the industry. And we can now conclude that this industry, as a component of ancestral Polynesian society, was no longer present by 2350 cal BP.

## Supporting information

S1 File Detailed data for individual radiocarbon dates. (DOCX)

S2 File Calculation of population growth in Tonga 2850–2350 cal BP. (DOCX)
