# Supplementary material for: Early cessation of ceramic production for ancestral Polynesian society in Tonga
Source: PLoS One. 2018 Feb 23;13(2):e0193166. doi: 10.1371/journal.pone.0193166 (PMC5825035; doi:10.1371/journal.pone.0193166)
Supplement: S1 File — (DOCX) [file pone.0193166.s001.docx]

**S1 File. Detailed Data for Individual Radiocarbon Dates**

Radiocarbon dates are calibrated in Oxcal using the southern hemisphere calibration curve SHCal13 [1]. δ^13^C is provided where measured independently and presented by the lab. Dates measured by University of Ottawa AMS laboratory (UOC) have δ^13^C measured on the AMS and contain machine fractionation. Two dates taken from earlier publications did not have δ^13^C reported, as is noted in comments.

***Tongatapu Radiocarbon Dates***

**Talasiu Site WK-28234. 2473 ± 31 BP δ^13^C= -21.3%**

Coconut endocarp, Shell Midden, 55-60cm below surface. Cal BP 2678-2360 at 68.2%; Cal BP 2701-2353 at 95.4% [2].

**Talasiu Site WK-28235. 2510 ± 30 BP δ^13^C= -21.3%**

Coconut endocarp, Shell Midden, 70-75cm below surface. Cal BP 2702-2465 at 68.2%; Cal BP 2715-2379 at 95.4% [2].

**Talasiu Site WK-22876. 2452 ± 30 BP δ^13^C= -23.5%**

Unidentified Wood Unidentified Wood Charcoal, Burial Structure. Cal BP 2486-2355 at 68.2%; Cal BP 2698-2346 at 95.4% [2]**.**

**Talasiu Site WK-23002. 2562 ± 30 BP δ^13^C= -25%**

Unidentified Wood Charcoal, Burial Structure. Cal BP 2733-2504 at 68.2%; Cal BP 2746-2486 at 95.4% [2].

**Talasiu Site WK-33572. 2553 ± 25 BP δ^13^C= -22.6%**

Coconut endocarp, Pit 2, Spit 4, Upper Strata. Cal BP 2721-2502 at 68.2%; Cal BP 2741-2487 at 95.4% [2].

**Talasiu Site WK-33573. 2448 ± 25 BP δ^13^C= -22.5%**

Coconut endocarp, Pit 2, Spit 11, Middle Strata. Cal BP 2465-2355 at 68.2%; Cal BP 2695-2346 at 95.4% [2].

**Talasiu Site WK-33574. 2504 ± 25 BP δ^13^C= -23%**

Coconut endocarp, Pit 2, Spit 18, Lower Strata. Cal BP 2700-2458 at 68.2%; Cal BP 2710-2376 at 95.4% [2].

**Ha’ateiho Site UOC-3859. 2583 ± 22 BP δ^13^C= N/A**

Coconut endocarp, Unit 4, Level 6, 70 cm below surface, associated with Mid-Plainware ceramic period occupation. Cal BP 2744-2539 at 68.2%; Cal BP 2749-2494 at 95.4%. New Date DVB.

**Ha’ateiho Site UOC-3860. 2452 ± 25 BP δ^13^C= N/A**

Unidentified Wood Charcoal, Unit 7, Level 6, 80 cm below surface, associated with Lapita/Plainware transition. Cal BP 2716-2498 at 68.2%; Cal BP 2738-2462 at 95.4%. New Date DVB.

**Ha’ateiho Site UOC-3861. 2499 ± 22 BP δ^13^C= N/A**

Unidentified Wood Charcoal, Unit 9, Level 5, 72 cm below surface, associated with Mid- to Late Plainware ceramic period occupation. Cal BP 2698-2434 at 68.2%; Cal BP 2705-2363 at 95.4%. New Date DVB.

**Ha’ateiho Site UOC-3862. 2493 ± 25 BP δ^13^C= N/A**

Unidentified Wood Charcoal, Unit 10, Level 3, 67 cm below surface, associated with Late Plainware ceramic period occupation. Cal BP 2696-2379 at 68.2%; Cal BP 2704-2360 at 95.4%. New Date DVB.

**Fakala’a Site S-ANU-54628. 2439 ± 38 BP δ^13^C= -22%**

Coconut endocarp, Test Pit 1, 47cm below surface, associated with Plainware ceramics amidst dense shell, Unidentified Wood Charcoal, worked coral, and oven stones. Cal BP 2531- 2487 at 68.2% Cal BP 2700-2431 at 95.4%**.** New Date GC.

**Moisa Site S-ANU-54629. 2461 ± 32 BP δ^13^C= -25%**

Coconut endocarp, Test Pit 1, 75cm below surface, associated with Plainware ceramics amidst dense shell and Unidentified Wood Charcoal. Cal BP 2676-2357 at 68.2% Cal BP 2700-2350 at 95.4%. *Comment*: Sample located in a small pocket of shell midden amongst basal deposits. New Date GC.

**Tufumahina Site NZ-636. 2380 ± 51 BP δ^13^C= N/A**

Unidentified Wood Charcoal. Cal BP 2460-2312 at 68.2%; Cal BP 2695-2163 at 95.4% [3]. *Comment:* δ^13^C not reported.

***Ha’apai Radiocarbon Dates – Lifuka Island***

**Tongoleleka Site UOC-3872. 2540 ± 24 BP δ^13^C= N/A**

Unidentified Wood Charcoal, Unit 11, Level 4, 36 cm below surface, associated with Mid- to Late Plainware ceramic period occupation. Cal BP 2715-2498 at 68.2%; Cal BP 2737-2462 at 95.4%. New Date DVB.

**Tongoleleka Site UOC-3873. 2550 ± 23 BP δ^13^C= N/A**

Unidentified Wood Charcoal, Unit 1, Level 5, 45 cm below surface, associated with Mid-Plainware ceramic period occupation. Cal BP 2720-2501 at 68.2%; Cal BP 2740-2486 at 95.4%. New Date DVB.

**Tongoleleka Site UOC-3874. 2460 ± 22 BP δ^13^C= N/A**

Unidentified Wood Charcoal, Unit 11, Level 9, 89 cm below surface, associated with Lapita/Plainware transition. Cal BP 2485-2359 at 68.2%; Cal BP 2696-2350 at 95.4%. New Date DVB.

**Tongoleleka Site CAMS-34558. 2450 ± 40 BP δ^13^C= -22.9%**

Unidentified Wood Charcoal, Unit 4, Level 5, 50 cm below surface, associated with Mid-Plainware ceramic period occupation. Cal BP 2676-2352 at 68.2%; Cal BP 2700-2346 at 95.4% [4], [5].

**Tongoleleka Site CAMS-34559. 2600 ± 60 BP δ^13^C= -27.4%**

Unidentified Wood Charcoal, Unit 4, Level 8, 75 cm below surface, associated with Early Plainware ceramic period occupation. Cal BP 2753-2500 at 68.2%; Cal BP 2780-2380 at 95.4% [4], [5].

**Tongoleleka Site CAMS-34560. 2560 ± 50 BP δ^13^C= -27.9%**

Unidentified Wood Charcoal, Unit 4, Level 10, 103 cm below surface, associated with Lapita/Plainware transition. Cal BP 2738-2494 at 68.2%; Cal BP 2750-2380 at 95.4% [4], [5].

**Tongoleleka Site CAMS-41512. 2490 ± 51 BP δ^13^C= -23.3%**

Coconut endocarp, Unit 11, Level 4, 38 cm below surface, associated with Mid- to Late Plainware ceramic period occupation. Cal BP 2696-2364 at 68.2%; Cal BP 2710-2357 at 95.4% [4], [5].

**Tongoleleka Site CAMS-41513. 2430 ± 50 BP δ^13^C= -26.4%**

Coconut endocarp, Unit 11, Level 7, 66 cm below surface, associated with Early to Mid-Plainware ceramic period occupation. Cal BP 2676-2346 at 68.2%; Cal BP 2702-2330 at 95.4% [4], [5].

**Tongoleleka Site BETA-14171. 2330 ± 60 BP δ^13^C= N/A**

Unidentified Wood Charcoal, Unit 45N1W, top of Layer III. Cal BP 2356-2178 at 68.2%; Cal BP 2487-2116 at 95.4% [6]. *Comment:* δ^13^C not reported.

**Holopeka Site CAMS-12919. 2590 ± 60 BP δ^13^C= -23.9%**

Unidentified Wood Charcoal, Unit 97 N/100 W, Level 9, 85 cm below surface, Lower Stratum, associated with Early Plainware ceramic period occupation. Cal BP 2750-2498 at 68.2%; Cal BP 2768-2380 at 95.4% [4], [5].

**Holopeka Site CAMS-41527. 2540 ± 50 BP δ^13^C= -9.98%**

Coconut endocarp, Unit 95 N/100 W, Level 5, 46 cm below surface, associated with Late Plainware ceramic period occupation. Cal BP 2722-2490 at 68.2%; Cal BP 2742-2379 at 95.4% [4], [5]. *Comment:* δ^13^C measurement of an unidentified C4 plant pathway**.**

**Holopeka Site CAMS-41528. 2510 ± 50 BP δ^13^C= -22.7%**

Unidentified Wood Charcoal, Unit 96 N/100 W, Level 11, 104 cm below surface, taken from post hole fill, associated with Late Plainware ceramic period occupation. Cal BP 2705-2460 at 68.2%; Cal BP 2717-2364 at 95.4% [4], [5].

***Ha’apai Radiocarbon Dates – ‘Uiha Island***

**Vaipuna Site CAMS-41523. 2580 ± 50 BP δ^13^C= -24.3%**

Unidentified Wood Charcoal, Unit 12, Level 5, 55 cm below surface, associated with Late Plainware ceramic period occupation. Cal BP 2745-2500 at 68.2%; Cal BP 2758-2434 at 95.4% [4], [5].

**Vaipuna Site CAMS-41525. 2560 ± 80 BP δ^13^C= -23.6%**

Unidentified Wood Charcoal, Unit 14, Level 5, 60 cm below surface, associated with Mid- to Late Plainware ceramic period occupation. Cal BP 2742-2490 at 68.2%; Cal BP 2752-2364 at 95.4% [4], [5].

***Ha’apai Radiocarbon Dates – Ha’ano Island***

**Pukotala Site CAMS-41515. 2560 ± 50 BP δ^13^C= -26.7%**

Unidentified Wood Charcoal, Unit 14, Level 9, 95 cm below surface, associated with Early Plainware ceramic period occupation. Cal BP 2738-2494 at 68.2%; Cal BP 2750-2380 at 95.4% [4], [5].

**Pukotala Site CAMS-41517. 2540 ± 50 BP δ^13^C= -22.9%**

Unidentified Wood Charcoal, Unit 12, Level 7, 70 cm below surface, associated with Mid-Plainware ceramic period occupation. Cal BP 2722-2490 at 68.2%; Cal BP 2742-2379 at 95.4% [4], [5].

***Ha’apai Radiocarbon Dates – Foa Island***

**Faleloa Site CAMS-7146. 2560 ± 60 BP δ^13^C= -25.3%**

Unidentified Wood Charcoal, Unit 10, Level 10, 95 cm below surface, associated with Lapita/Plainware transition. Cal BP 2740-2492 at 68.2%; Cal BP 2750-2379 at 95.4% [4], [5].

**Faleloa Site CAMS-41529. 2550 ± 50 BP δ^13^C= -22.7%**

Coconut endocarp, Unit 18, Level 3, 45 cm below surface, associated with Late Plainware ceramic period occupation. Cal BP 2731-2492 at 68.2%; Cal BP 2746-2380 at 95.4% [4], [5].

***Ha’apai Radiocarbon Dates – Ha’afeva Island***

**Mele Havea Site UOC-3866. 2493 ± 22 BP δ^13^C= N/A**

Unidentified Wood Charcoal, Unit 2, Level 5, 51 cm below surface, associated with Mid-Plainware ceramic period occupation. Cal BP 2696-2379 at 68.2%; Cal BP 2704-2360 at 95.4%. New Date DVB.

**Mele Havea Site UOC-3867. 2478 ± 22 BP δ^13^C= N/A**

Unidentified nut, Unit 3, Level 6, 65 cm below surface, associated with Lapita/Plainware transition. Cal BP 2677-2365 at 68.2%; Cal BP 2700-2356 at 95.4%. New Date DVB.

**Mele Havea Site UOC-3868. 2505 ± 22 BP δ^13^C= N/A**

Coconut endocarp, Unit 8, Level 6, 65 cm below surface, associated with Lapita/Plainware transition. Cal BP 2700-2460 at 68.2%; Cal BP 2710-2378 at 95.4%. New Date DVB.

**Mele Havea Site UOC-3869. 2491 ± 22 BP δ^13^C= N/A**

Coconut endocarp, Unit 10, Level 6, 65 cm below surface, associated with Lapita/Plainware transition. Cal BP 2692-2379 at 68.2%; Cal BP 2702-2360 at 95.4%. New Date DVB.

**Mele Havea Site CAMS-41519. 2490 ± 50 BP δ^13^C= -23.2%**

Coconut endocarp, Unit 3, Level 5, 55 cm below surface, associated with Mid-Plainware ceramic period occupation. Cal BP 2696-2364 at 68.2%; Cal BP 2710-2357 at 95.4% [4], [5].

**Mele Havea Site CAMS-41521. 2510 ± 50 BP δ^13^C= -24.4%**

Unidentified Wood Charcoal, Unit 10, Level 5, 53 cm below surface, associated with Mid-Plainware ceramic period occupation. Cal BP 2705-2460 at 68.2%; Cal BP 2717-2364 at 95.4% [4], [5].

***Vava’u Radiocarbon Dates***

**Otea Site UOC-3863. 2529 ± 29 BP δ^13^C= N/A**

Coconut endocarp, Unit 12, Level 14, 145 cm below surface, associated with Late Plainware ceramic period occupation. Cal BP 2712-2492 at 68.2%; Cal BP 2734-2380 at 95.4%. New Date DVB.

**Otea Site UOC-3865. 2572 ± 26 BP δ^13^C= N/A**

Unidentified Wood Charcoal, Unit 6, Level 15, 152 cm below surface, associated with Late Plainware ceramic period occupation. Cal BP 2740-2510 at 68.2%; Cal BP 2748-2490 at 95.4%. New Date DVB.

**Falevai Site UOC-3870. 2483 ± 22 BP δ^13^C= N/A**

Unidentified Wood Charcoal, Unit 3, Level 15, 150 cm below surface, associated with Lapita/Plainware transition. Cal BP 2682-2376 at 68.2%; Cal BP 2700-2356 at 95.4%. New Date DVB.

**Falevai Site** **UOC-3871. 2561 ± 25 BP δ^13^C= N/A**

Unidentified Wood Charcoal, Unit 13, Level 15, 150 cm below surface, associated with Lapita/Plainware transition. Cal BP 2730-2504 at 68.2%; Cal BP 2744-2489 at 95.4%. New Date DVB.

**Falevai Site CAMS-119694. 2500 ± 35 BP δ^13^C= -27.9%**

Unidentified Wood Charcoal, Unit 4, Level 11, 105 cm below surface, associated with Mid-Plainware ceramic period occupation. Cal BP 2700-2433 at 68.2%; Cal BP 2708-2363 at 95.4% [5], [7].

**Falevai Site CAMS-119695. 2645 ± 35 BP δ^13^C= -28.2%**

Unidentified Wood Charcoal, Unit 9, Level 15, 145 cm below surface, associated with Lapita/Plainware transition. Cal BP 2765-2718 at 68.2%; Cal BP 2791-2502 at 95.4% [5], [7].

**S1 File References**

1 Hogg A, Hua Q, Blackwell P, Niu M, Buck C, Guilderson T et al. SHCal13 southern hemisphere calibration, 0–50,000 years cal BP. Radiocarbon. 2013; 55(4): 1889–1903

2 Clark G, Grono E, Ussher E, Reepmeyer C. Early settlement and subsistence on Tongatapu, Kingdom of Tonga: Insights from a 2700-2650 cal BP midden deposit. J Archaeol Sci Reps. 2016; 3: 513-524.

3 Spennemann, DHR. ‘Ata ‘a Tonga mo ‘ata ‘o Tonga: Early and later prehistory of the Tongan Islands. Ph.D. Thesis, Australian National University, Canberra. 1989.

4 Burley DV, Nelson E, Shutler R. A radiocarbon chronology for the Eastern Lapita Frontier in Tonga. Archaeol Oceania. 1999; 34: 59–72.

5 Burley D, Edinborough K, Weisler M, Zhao J-X. Bayesian modeling and chronological precision for Polynesian settlement of Tonga. PLoS One. 2015; 10(3): e120795. https://doi.org/ /10.1371/journal.pone.0120795

6 Dye T. Early eastern Lapita to Polynesian plainware at Tongatapu and Lifuka: An exploratory data analysis. In Davidson J, Irwin G, Leach F, Pawley A, Brown D, editors. Oceanic culture history: Essays in honour of Roger Green. Dunedin: NZ J Archaeol Special Publication; 2016. pp 461-473.

7 Burley DV, Connaughton SP. First Lapita settlement and its chronology in Vava’u, Kingdom of Tonga. Radiocarbon. 49(1): 131-137.
