# Supplementary material for: Early cessation of ceramic production for ancestral Polynesian society in Tonga
Source: PLoS One. 2018 Feb 23;13(2):e0193166. doi: 10.1371/journal.pone.0193166 (PMC5825035; doi:10.1371/journal.pone.0193166)
Supplement: S2 File — (DOCX) [file pone.0193166.s002.docx]

**S2 File. Calculation of Population Growth in Tonga 2850-2350 cal BP**

Populations associated with the Lapita and Polynesian Plainware phases in Tonga are assumed to be unconstrained by density dependent controls and population growth is exponential. The size of a Lapita founder population can only be speculated but we use the number 100 with expectations of four canoes carrying 25 individuals on each. The exponential growth formula as below is then applied to the 500-year period from the initial founder colony (2850 cal BP) to the end of the Polynesian Plainware phase (2350 cal BP):

*Xt* = *X*_0_ (1 + *r*)*^t^*

*Xt* = population estimate at end of Polynesian Plainware phase with elapsed time of 500 years

*X*_0_ = population size of founder colony (100)

*r* = population growth rate (either 0.0052 or 0.00875)

*t* = elapsed time (500 years)

Population estimate based on Hassan’s [1] maximum growth rate of 0.0052 is 1337 individuals at end of the Polynesian Plainware phase with a population doubling time of 133.5 years.

Population estimates based on pre-contact Maori projection [2] of 0.00875 is 7794 individuals at end of the Polynesian Plainware phase, with population doubling time of 79.6 years.

Estimates have not taken into consideration the possibilities for additional immigration or emigration for the periods in question.

**S2 File References**

1 Hassan FA. Demographic archaeology. New York: Academic Press; 1981.

2 Brewis AA, Molloy M, Sutton DG. Modeling the prehistoric Maori population, Am J Phys Anthropol. 1990; 81: 343-356.
